# Dietary Supplementation with Low-Molecular-Weight Fucoidan Enhances Innate and Adaptive Immune Responses and Protects against *Mycoplasma pneumoniae* Antigen Stimulation

**DOI:** 10.3390/md17030175

**Published:** 2019-03-18

**Authors:** Pai-An Hwang, Hong-Ting Victor Lin, Hsin-Yuan Lin, Szu-Kuan Lo

**Affiliations:** 1 Department of Bioscience and Biotechnology, National Taiwan Ocean University, No. 2, Beining Road, Keelung 20246, Taiwan; 2063B003@mail.ntou.edu.tw; 2 Department of Food Science, National Taiwan Ocean University, Keelung 20246, Taiwan; HL358@ntou.edu.tw; 3 Gi-Kang Clinic, No. 155, Yanping Rd., Zhongli Dist., Taoyuan 32043, Taiwan; a912164@yahoo.com.tw

**Keywords:** low molecular weight fucoidan, *Mycoplasma pneumoniae*, NK cell, antigen-specific antibody, adjuvant

## Abstract

In this study, the low-molecular-weight (LMW) fucoidan, rich in fucose and sulfate, was extracted and purified from the edible brown seaweed, *Laminaria japonica*. In this study, we orally administered LMW fucoidan to mice for 6 weeks. We then examined fucoidan’s effects on innate immunity, adaptive immunity, and *Mycoplasma pneumoniae* (MP)-antigen-stimulated immune responses. Our data showed that LMW fucoidan stimulated the innate immune system by increasing splenocyte proliferation, natural killer (NK) cell activity, and phagocytic activity. LMW fucoidan also increased interleukin (IL)-2, IL-4, and interferon (IFN)-γ secretion by splenocytes and immunoglobulin (Ig)-G and IgA content in serum, which help regulate adaptive immune cell functions, and decreased allergen-specific IgE. In MP-antigen-stimulated immune responses, the IgM and IgG content in the serum were significantly higher in the LMW fucoidan group after MP-antigen stimulation. Our study provides further information about the immunomodulatory effects of LMW fucoidan and highlights a potential role in preventing *M. pneumoniae* infection.

## 1. Introduction

Before the 1950s, seaweeds were used as traditional folk medicines [1]. Over the past two decades, polysaccharides of brown seaweeds, such as fucoidan, alginate, and laminarin, have been investigated for their biological activities [2,3]. The health-promoting effects of these compounds, especially fucoidan, give brown seaweed great value for developing natural dietary supplements.

Fucoidans are a class of fucose-containing sulfated polysaccharides found in brown seaweed [4]. Most fucoidans have complex chemical compositions, and their structures and biological activities vary from species to species [5]. Its non-animal origin has been related to particular pharmacological activities [6]. Fucoidan has been associated with antitumor [7,8,9], antiviral [10], anti-inflammatory [11,12], anticoagulant [13], and osteogenic-enhancing differentiation activities [14]. These activities are closely related to its molecular weight [15] and sulfate content [16].

Fucoidan’s immunomodulatory effects have been reported in different experimental models. It has been shown to activate and promote the maturation of human monocyte-derived dendritic cells [17], and stimulate lymphocyte [18,19] and macrophage activity [18,20] in vitro. According to Do et al. [21], fucoidan is an immunomodulating nutrient which alters the sensitivity of glia and macrophages. Jin et al. [22] found that intraperitoneal (i.p.) fucoidan injection in mice upregulated CD40, CD80, and CD86 expression. It also increased production of interleukin (IL)-6, IL-12, and tumor necrosis factor-α (TNF-α) in spleen dendritic cells (DC). Furthermore, fucoidan promoted interferon (IFN)-γ generation by producing Th1 and Tc1 cells in an IL-12-dependent manner. Zhang et al. [23] also showed that i.p. administration of fucoidan in the mice promoted natural killer (NK) cell, DC, and T-cell activation. In addition, when mice are injected IP with ovalbumin (OVA) and fucoidan, mice were found to produce remarkably higher amounts of anti-OVA Immunoglobulin G (IgG) than OVA-control mice, and fucoidan was found to promote the generation of effector/memory T-cells based on the surface expression of CD44. Intravenous administration of fucoidan upregulated CD40, CD80, CD86, and the major histocompatibility complex in mouse spleen DCs [23]. Interestingly, oral administration of 300 mg fucoidan per day to elderly men and women increased their immune responses to influenza vaccination [24], but the underlying mechanisms of this are still unclear.

There are some reports on the relationship between the structure and immunomodulatory properties of fucoidan [25]; however, less is known about the relationship between their molecular weight and immunomodulatory properties. In our previous studies, we demonstrated that low-molecular-weight (LMW) fucoidan has more favorable bioactivity in vitro and in vivo than high-molecular-weight fucoidan [7,8,9,12], with no toxicological effects found in rats after 28 days of repeated oral administration [26]. LMW fucoidan is expected to be a safe food supplement for immunomodulation.

*Mycoplasma pneumoniae* is one of the most common agents of community-acquired pneumonia in previously healthy people, and infection can occur at any age [27]. *M. pneumoniae* is considered a self-limiting disease, but some patients suffer from protracted and complicated courses [28]. In addition, *M. pneumoniae* can change its cell membrane composition to mimic the host’s cell membrane, thus avoiding immune system detection [27]. Currently, there is no vaccine to prevent *M. pneumoniae* infection. Macrolide is one of the first-line drugs used to treat *M. pneumoniae*, but the prevalence of macrolide-resistant *M. pneumonia* is increasing worldwide. In 2011, the macrolide-resistance rate was up to 23% in Taiwan [29]. A recent study has reported that early additional immune-modulators might can prevent *M. pneumoniae* disease progression and reduce morbidity [30]. A natural and safe food supplement that enhances the immune response to prevent *M. pneumoniae* infection could address this unmet need.

In this study, we evaluated the immunomodulatory effects of orally administered LMW fucoidan on innate and adaptive immune responses in mice. We also evaluated the effect of LMW fucoidan supplementation on immune responses to the *M. pneumonia* (MP) antigen.

## 2. Results and Discussion

### 2.1. Fractionation and Composition of Crude Fucoidan and LMW Fucoidan

**Figure 1:**
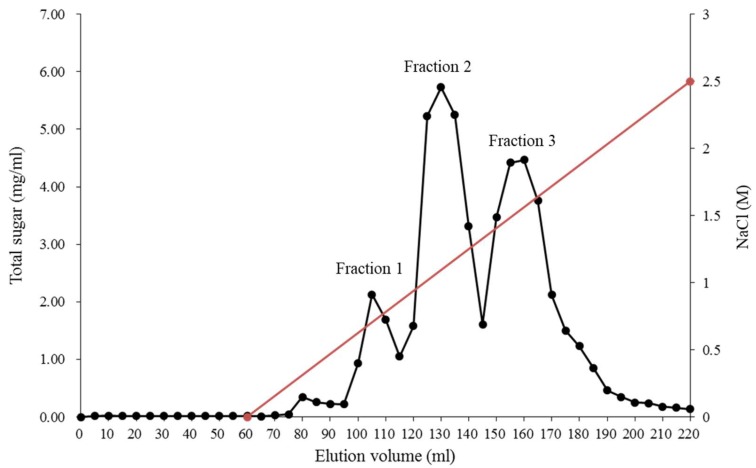
Fractionation of crude fucoidan isolated from the *L. japonica* on a DEAE (Diethylaminoethyl)-Sephadex A-25 column.

A step gradient was used to selectively elute the sulfate and fucose-rich fucoidan from a crude brown seaweed extract. The yields and compositions of the three fractions, purified from *Laminaria japonica*, are shown in Figure 1 and Table 1.

The fucoidan fraction 1, which was eluted at the lowest NaCl concentration, was rich in galactose (35.8 ± 0.5% mol) and glucose (20.6 ± 0.2% mol), but had low concentrations of fucose (12.9 ± 0.2% mol) and sulfate (10.6 ± 0.6%). The fucoidan fraction 3, eluted at a higher concentration of NaCl, was rich in fucose (48.2 ± 0.3% mol) and sulfate (39.5 ± 0.8%). Although the fucoidan fraction 2 had the highest yield, the fucose (34.6 ± 0.4% mol) and sulfate (25.2 ± 0.5%) contents were lower than in fraction 3. Fucoidan fractions presenting diversities in their sulfation degrees and patterns also differed in their biological activities [31,32]. Thus, we selected fraction 3 as the purified fucoidan and hydrolyzed it as our LMW fucoidan sample. The fraction 3 was hydrolyzed with glycolytic enzyme preparation to obtain the LMW fucoidan sample with a molecular weight lower than 3000 Da. The LMW fucoidan consisted of 40.5 ± 0.8% mol fucose, 5.7 ± 0.7% mol glucose, 28.3 ± 0.8% mol galactose, 5.4 ± 0.5% mol myo-inositol, 15.6 ± 0.5% mol mannose, 3.3 ± 0.6% mol xylose, 1.2 ± 0.4% mol rhamnose, and its degree of sulfation was 31.4 ± 1.6% (Table 1). Myo-inositol has been reported to be the minor sugars in brown seaweed, such as *Fucus vesiculosus* [33], *Himanthalia elongate* [34], and *Bzjizrcaria bifurcate* [35]. Tarakhovskaya et al. [33] indicated that the myo-inositols might have several biological functions, which serve as an initial precursor of different cell-wall polysaccharides.

### 2.2. Effects of LMW Fucoidan on Innate Immune Responses in Mice

Innate immunity is the first line of defense against almost any substance that threatens the body. Poor environmental factors, such as malnutrition, stress, and wake-sleep disorders may cause innate immune system deficiencies. Functional nutrient studies indicate a positive correlation between the innate immune system and health [36]. Here, we first examined the effects of oral LMW fucoidan on the innate immune response of non-immunized mice.

It was shown in our previous study that fucoidan was not toxic after intragastric administration to Sprague Dawley (SD) rats at 2000 mg/kg/day [26]. Anti-diabetes activity of LMW fucoidan was observed at the dose of 300–600 mg/kg/day in C57BLKS/J Iar-+Leprdb/+Leprdb (db/db) mice [37]. As a result, dosages of 200, 600, and 1000 mg/kg was chosen in this study for mice.

#### 2.2.1. Spleen Weight and Proliferative Responses of Splenocytes

**Figure 2:**
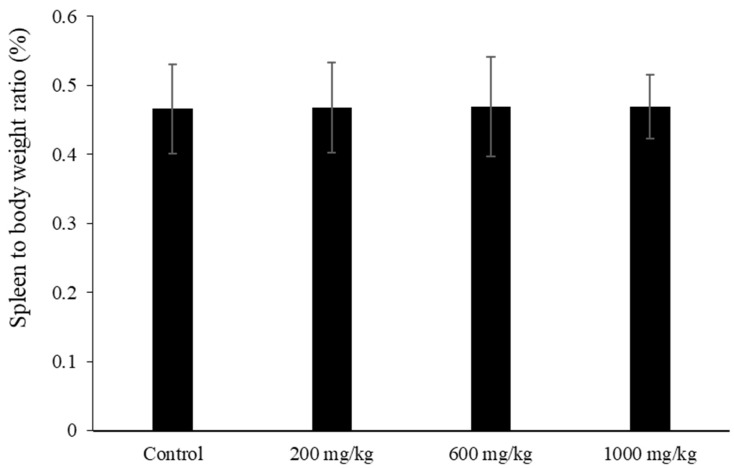
The spleen-to-body-weight ratios of mice treated with low-molecular-weight (LMW) fucoidan for 6 weeks. Spleen-to-body-weight ratio = [spleen weight (g)/body weight (g)] × 100%. Data were expressed as mean ± SD of ten mice.

The spleen plays an important role in host defense, and is the site for innate and adaptive immune processes. We first examined spleen weight and the proliferative response of splenocytes in LMW fucoidan-treated mice. Mice were orally administered 200, 600, and 1000 mg/kg LMW fucoidan or distilled water (control group) daily for 6 weeks, and then sacrificed. During the 6 weeks, a steady rise in body weight (from 17.5 ± 0.2 g to 20.4 ± 0.3 g) was observed, and there were no statistically significant differences between the treated and control mice. The spleen weights were obtained post-mortem, and the spleen-to-body-weight ratio was calculated. No significant differences were observed in the spleen-to-body-weight ratio among the four groups (Figure 2).

This result is consistent with a previous study showing that feeding mice fucoidan from *Fucus vesiculosus* had no effects on the spleen-to-body-weight ratio [38]. In addition, Jang et al. [39] showed that LMW fucoidan was less toxic to spleen cells than high-molecular-weight fucoidan. Therefore, LMW fucoidan is not a sensitive indicator to cause cell stress or immunotoxicity. After harvesting spleen cells, splenocytes were prepared and stimulated with the T-cell mitogen concanavalin A (Con A) or B-cell mitogen lipopolysaccharides (LPS) for 72 h. Splenocytes from mice fed with 200, 600, and 1000 mg/kg LMW fucoidan showed significantly increased Con A and LPS-stimulated proliferation compared to the control mice (Table 2), indicating that LMW fucoidan had increased both T- and B-cell proliferation. This is consistent with the results of previous studies by Jang et al. [39], who reported that both low- and high-molecular-weight fucoidan increase spleen-cell viability. Our results confirm that LMW fucoidan has immunostimulatory effects.

#### 2.2.2. Natural Killer (NK) Cell Activity

NK cells are a component of the innate immune system, which plays an important role in the early stages of tumor cell elimination. Previous research has shown that NK cell activity is relatively sensitive to diet and the intake of specific food components [40]. To investigate LMW fucoidan’s effect on enhancing NK cell activity, we assessed whether LMW fucoidan could induce cytotoxic NK cell activity against YAC-1 cells which are sensitive to lysis by activated NK cells. Our results showed that NK cell activity was enhanced in mice receiving 200, 600, and 1000 mg/kg LMW fucoidan compared to controls (Table 3). Namkoong et al. [41] and Maruyama et al. [42] also reported that fucoidan can increase NK cell activity via oral administration. Together, these data indicate that LMW fucoidan consumption can activate NK cells and, thus, might be a good way of enhancing the immune system.

#### 2.2.3. Phagocytic Activity of Peritoneal Cells

As part of the innate immune system, the body has developed defenses mediated by specialized cells which destroy invading microorganisms by ingestion and phagocytosis. In the present study, the phagocytic activity of peritoneal cells from mice which were fed LMW fucoidan was measured by quantifying internalized Fluorescein isothiocyanate (FITC)-*Escherichia coli* (*E. coli*). Cells isolated from mice treated with 1000 mg/kg LMW fucoidan exhibited a significant increase in phagocytosis at multiplicities of infection of 12.5 and 25 (Table 4). These results are similar to those reported by Anisimova et al. [43], who showed that fucoidan intensifies the engulfment of microorganisms by human blood, increasing both the relative number and efficiency of phagocytes.

In summary, we found that LMW fucoidan effectively stimulates innate immunity, not only by phagocytosis (Table 4) but also by activating extracellular killing by NK cells (Table 3) to destroy invading pathogens.

### 2.3. Effects of LMW Fucoidan on Adaptive Immune Responses (OVA-Specific Immunity) in Mice

The above results prompted us to further investigate the adjuvant effects of LMW fucoidan on adaptive immune responses. We orally administered LMW fucoidan daily for 6 weeks and immunized mice with OVA to examine the effect on cytokine response and specific antibody production against OVA.

#### 2.3.1. Proliferative Response by Con A, LPS, and OVA Stimulation

**Figure 3:**
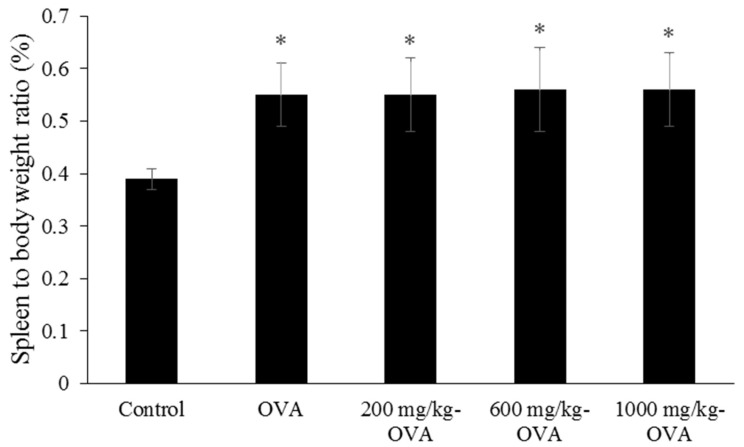
The spleen-to-body-weight ratios of ovalbumin (OVA)-immunized mice treated with LMW fucoidan for 6 weeks. Spleen-to-body-weight ratio = [spleen weight (g)/body weight (g)] × 100%. Data were expressed as mean ± SD of ten mice. Means with asterisks were significantly different from the control (*p* < 0.05).

Mice were orally administered distilled water (control group) or 200, 600, and 1000 mg/kg LMW fucoidan daily for 6 weeks, and OVA immunization was conducted at the third and fifth weeks. During the 6 weeks, a steady rise in body weight (from 18.6 ± 0.3 g to 21.4 ± 0.4 g) was observed, and there were no statistically significant differences between the groups of mice. The spleen weights were obtained, and the spleen-to-body-weight ratio was calculated. Interestingly, the ratios of OVA-immunized and OVA-immunized + LMW fucoidan-treated mice were higher than those of controls (Figure 3), although feeding LMW fucoidan alone for 6 weeks did not alter the ratio (Figure 2).

A previous study reported that the spleen-to-body-weight ratio of LPS + fucoidan-treated mice was higher than LPS-treated mice when fucoidan was administered intraperitoneally [38], suggesting that different routes of fucoidan administration may lead to significantly different physiological reactions. Our findings suggest that OVA plays a major role in increasing the spleen-to-body-weight ratio, and that oral administration of LMW fucoidan does not support this effect. LMW fucoidan (200, 600, and 1000 mg/kg doses) significantly enhanced the mitogen (Con A and LPS)- and OVA-stimulated splenocyte proliferation in OVA-immunized mice compared to the OVA-control group (Table 5). These findings indicate that LMW fucoidan could significantly increase cell-mediated immune responses in OVA-immunized mice.

#### 2.3.2. Cytokine Secretion by OVA-Stimulation

Cytokines serve as chemical messengers within the immune system and are primarily produced by T-helper (Th) cells. In the activation phase of adaptive immune responses, cytokines stimulate the growth and differentiation of lymphocytes. In the effector phases, they activate different effector cells to eliminate microbes and other antigens [44]. We examined the effect of LMW fucoidan on the production of cytokines that mediate and regulate adaptive immune responses. Th 1 cytokines, including IL-2 and IFN-γ, and the Th 2 cytokine, IL-4, significantly increased following LMW fucoidan administration. No significant differences in the production of the Th2 cytokine IL-5 were observed. LMW fucoidan treatment during OVA-stimulation significantly decreased TNF-α, a multifunctional, pro-inflammatory cytokine (Table 6). NK cell activity has been shown to be enhanced by IL-2 [45], IL-4 [46], and IFN-γ [47], and our data also showed that IL-2, IL-4, and IFN-γ production (Table 6) and NK cell activity (Table 3) were increased in splenocytes from mice treated with oral LMW fucoidan. Both Jin et al. [22] and Zhang et al. [23] reported that IP injection of fucoidan led to increased IFN-γ production compared to control mice immunized with OVA alone. These results indicate that fucoidan, administered either orally or via IP injection, could function as an adjuvant by promoting Th1-type immune responses.

The Th1/Th2 concept suggests that regulating the relative contribution of Th1- or Th2-type cytokines will likely modulate the development and strength of immune responses [48]. A previous study reported that ingesting brown seaweed enhanced OVA-specific Th1 and Th2 cytokine responses by draining lymph nodes in mice [49]. Similarly, our results showed that both the Th1 and Th2 responses in OVA-stimulated mice significantly increased after oral administration of LMW fucoidan. Together, these results demonstrate that oral LMW fucoidan stimulates a healthy Th1/Th2 balance.

#### 2.3.3. Serum Immunoglobulins Levels

Immunoglobulins, also known as antibodies, are glycoprotein molecules produced by plasma cells that protect us from pathogen infections and antigen sensitization. Immunoglobulin M (IgM) antibodies play an important role in the early stages of immune surveillance. Immunoglobulin A (IgA) is a major humoral factor of mucosal immunity, and IgE is an allergen-specific immunoglobulin that is stimulated by different allergens to produce the corresponding IgE [50]. We examined the effect of LMW fucoidan on the serum levels of anti-OVA IgG, anti-OVA IgA, and anti-OVA IgE that mediate adaptive immune responses. As expected, immunization with OVA significantly increased the serum levels of anti-OVA IgG, anti-OVA IgA, and anti-OVA IgE compared to control mice. Anti-OVA IgG and anti-OVA IgA were significantly increased in the serum from mice administered with LMW fucoidan compared with OVA-immunized mice (Table 7). Similar results were found in the production of IL-2 and IL-4, which stimulate B-cell activation and differentiation [51]. These data suggested that LMW fucoidan functions as an adjuvant to enhance antigen-specific immune responses. Furthermore, in these mice, anti-OVA IgE was significantly decreased (Table 7), so LMW fucoidan might be useful in counteracting allergic responses. Collectively, LMW fucoidan had a significant effect on cytokine expression by enhancing the production of IL-2, IL-4, and IFN-γ (Table 6). Thus, it is possible that LMW fucoidan favors Th1 differentiation and decreases IgE [52].

### 2.4. Effects of LMW Fucoidan on MP-Antigen-Stimulated Immune Responses in Mice

According to the above data, LMW fucoidan showed strong immunomodulatory effects in innate and adaptive immunity. As shown in Table 2; Table 3, the mice treated with LMW fucoidan showed better proliferative responses and NK cell activity, and there were no significant differences between the 200, 600, and 1000 mg/kg groups. As shown in Table 4, the mice treated with LMW fucoidan showed better phagocytic activity, and the 600 and 1000 mg/kg groups exhibited the best activities (Table 4). As shown in Table 5 and Table 6, the mice treated with LMW fucoidan showed better proliferative responses and cytokines secretion, and there were no significant differences between the 200, 600, and 1000 mg/kg groups (except IL-2 in Table 6). As a result, we chose the 600 mg/kg dose concentration to investigate the effects of orally administered LMW fucoidan on MP antigen immunogenicity.

**Figure 4:**
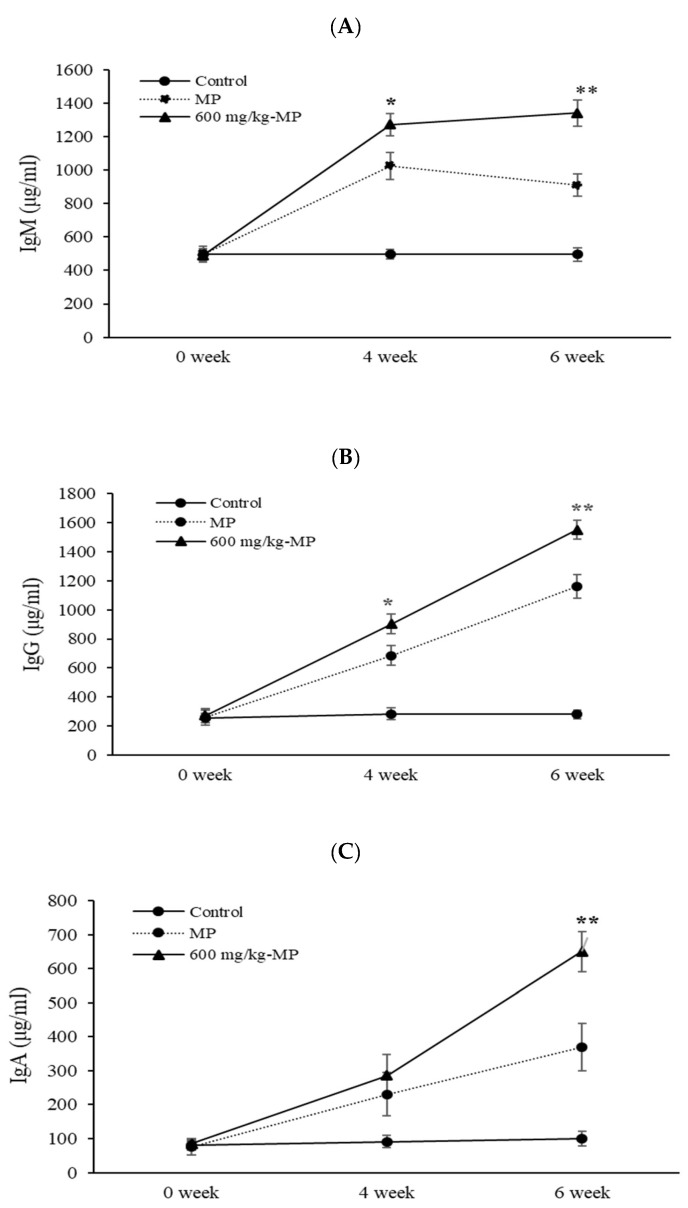
The IgM (**A**), IgG (**B**), and IgA (**C**) productions of *Mycoplasma pneumoniae* (MP) antigen-inoculated mice treated with 600 mg/kg LMW fucoidan for 6 weeks. Data were expressed as mean ± SD of ten mice. Values are expressed as mean ± SD. Means with asterisk were significantly different from the other groups (*p* < 0.05).

This evaluation was done to determine whether LMW fucoidan could function as an early additional immunomodulator, preventing *M. pneumoniae* disease infection and reducing disease morbidity. In this experiment, MP antigen inoculation was carried out twice. The first MP antigen inoculation was considered the first infection by *M. pneumoniae*, and the second MP antigen inoculation was considered the re-infection. As expected, after the MP antigen inoculation, IgM, IgG, and IgA contents in the serum were higher in the LMW fucoidan group than in the control group. IgM and IgG significantly increased (*p* < 0.05) in the serum from 600 mg/kg LMW fucoidan + MP-antigen-inoculated group mice compared to the MP-antigen-inoculated group mice after both the first and second inoculations. There was no significant difference in IgA with or without LMW fucoidan after MP antigen inoculation (Figure 4). Our data show a possible adjunctive role of LMW fucoidan by stimulating antibody production upon repeat infection of *M. pneumoniae*. Fucoidan has also been reported to increase immune responses to seasonal influenza vaccinations [24] and improve *M. pneumoniae* vaccine efficacy [40]. Based on our previous research and these results, we suggest that LMW fucoidan might help prevent the spread of *M. pneumoniae*.

In this study, we investigated the immunomodulatory effects of orally administrated LMW fucoidan on innate immunity, adaptive immunity, and MP-antigen-stimulated immune responses. Our results indicate that LMW fucoidan can increase splenocyte proliferation, NK cell activity, and phagocytic activity, as well as IL-2, IL-4, and IFN-γ production. In addition, LMW fucoidan enhanced antigen-specific antibody production in OVA- and MP-antigen-stimulated mice. LMW fucoidan, in the form of a natural food supplement, could enhance immune responses and attenuate the effects of *M. pneumoniae* infection.

## 3. Materials and Methods

### 3.1. Animal

The female BALB/c mice (Albino mice maintained by Halsey Bagg) were obtained from BioLASCO Taiwan CO., Ltd (Taipei, Taiwan). All mice were weighted and given provisional numbers upon arrival. The animals were subjected to acclimation in a housing facility for at least eight days before starting the experiment. Only healthy mice were selected for use in the study. The mice were eight weeks of age at the start of the experiment, and were housed in a normal, environmentally controlled animal room with free access to pathogen-free feed and water ad libitum. These mice were randomly divided, with ten mice in each group.

### 3.2. LMW Fucoidan Preparation

LMW fucoidan extracted from *Laminaria japonica* were collaboratively manufactured by Hi-Q Marine Biotech International Ltd. (New Taipei City, Taiwan). Seaweed samples were ground to flour with a miniblender, and then dried with a dryer at 50 °C. The 100 g dried seaweed was treated with 5 L of distilled water and boiled at 100 °C for 30 min, and the extract was centrifuged at 10,000× *g* for 20 min. The supernatant was added with 4 M CaCl_2_ incubated for 1 h to separate alginic acid, and re-centrifuged at 10,000× *g* for 20 min. All polysaccharides were dialyzed (cut off 10,000 Da) using deionized water for 48 h, and precipitated by the addition of ethanol at the ratio of 1:3 (V/V) and left overnight to give the crude fucoidan. The fucoidan so obtained was fractionated by anion-exchange chromatography using DEAE-Sephadex A-25 (Cl^−^ form, Pharmacia, Uppsala, Sweden) using gradually increasing concentrations of sodium chloride (0–2.5 M) at a flow rate of 1 mL/min, and eluents (5 mL/tube) were separately collected. Each fraction was analyzed for sulfate content (Section 3.3) and monosaccharide composition (Section 3.4). The fraction of rich sulfate and fucose content (fraction 3) was extracted and hydrolyzed with a mixture of crude glycolytic enzymes, isolated from *Bacillus subtilis* by our lab (Section 3.5). The reaction mixture was passed through a filter (3000 Da cut off) by using Amicon Stirred Cells (Merck Germany) to collect the LMW fucoidan with a molecular weight lower than 3000 Da. The hydrolysis reaction of the fucoidan by the crude glycolytic enzymes was performed at pH 6.5, 37 °C for 24 h and heated at 95 °C for 20 min for inactivation of the enzyme.

### 3.3. Analysis of Degree of Sulfation

Sulfate content was determined according to the gelatinbarium method [53] using sodium sulfate (1 mg/mL) as a standard after acid hydrolysis of the polysaccharide extract sample in 6 N HCl at 100 °C for 6 h.

### 3.4. Monosaccharide Composition Analysis

The monosaccharides compositions of the eluted fractions from anion-exchange chromatography were separated by a High-performance anion-exchange chromatography (HPAEC) (Dionex BioLC system) using an anion-exchange column (DionexTM Carbopac PA-10, 4 × 250 mm, Dionex, Thermo Fisher Scientific, Waltham, MA, USA). The analysis of monosaccharides was carried out at an isocratic NaOH concentration of 18 mM at ambient temperature [54].

### 3.5. Preparation of the Crude Enzyme Mixtures from B. subtilis Cultures

One colony of B. subtilis, isolated from natto in Keelung local market, was cultivated in tryptic soy broth (TSB, Becton, Dickinson and Company, Sparks, MD, USA) at 30 °C, 150 rpm for 24 h. The cell cultures were centrifuged to remove bacterial cells to obtain the mixtures of extracellular glycosidic enzymes.

### 3.6. Animal Experiments

The LMW fucoidan was a light brownish-white powder, and well-soluble below the highest concentration. In this study, three independent experiments were undertaken to evaluate the innate immune, adaptive immune, and MP antigen-inoculated immune response, respectively. The first experiment was performed to assay innate immune, mice were assigned to four groups (n = 10); concentrations of 200, 600, and 1000 mg/kg LMW fucoidan were dissolved in distilled water and then administered by oral gavage in 10 mL/kg on a daily basis for 6 weeks. The control group (0 mg/kg LMW fucoidan) mice were treated identically with equal volumes of distilled water also via oral gavage throughout the study. Mice were weighted and sacrificed, and blood samples were collected at the end of the experiment for serum immunoglobulins assay. Spleens were collected and weighted, and the splenocytes were analyzed for cell proliferation, the cytokines secretion assay, and natural killer cell activity assay. Peritoneal cells were collected form mice abdominal cavity for the phagocytic activity assay. Each mouse was weighted once a week during the study period.

The second experiment was performed byadaptive immune mice assay, and five groups of mice (control, OVA-immunized, 200, 60,0 and 1000 mg/kg LMW fucoidan, then OVA-immunized) were orally administered distilled water or LMW fucoidan for 6 weeks. The first OVA IP immunization was conducted after 3 weeks of administration. 6.25 μg OVA was emulsified in the complete Freund’s adjuvant (CFA) and intraperitoneally injected into the mice. Two weeks after the first OVA immunization, mice were given a second injection of 12.5 μg OVA emulsified with incomplete Freund’s adjuvant (IFA) in order to enhance the OVA-specific immune responses. Blood samples were collected before the OVA inducement at the end of the experiment for the serum immunoglobulins assay. Mice were then sacrificed by isoflurane inhalation. Spleens were collected and weighted, and the splenocytes were analyzed for cell proliferation and the cytokines secretion assay.. Each mouse was weighted once a week during the study period.

The third experiment was performed to assay MP antigen-inoculated immune responds, three groups of mice (control, MP antigen-stimulation and 600 mg/kg LMW fucoidan then MP antigen stimulation) were orally administered distilled water or LMW fucoidan for 6 weeks. First, MP antigen (10 μg/mouse, MP901, Native Antigen, UK) IP stimulation was conducted after 3 weeks of administration. Two weeks after the first stimulation, mice were given a second injection of MP antigen (10 μg/mouse) [40]. Blood samples were collected before the first MP antigen stimulation, after one week of stimulation, and at the end of the experiment for the serum immunoglobulins assay. Each mouse was weighted once a week during the study period.

### 3.7. Splenocyte Proliferation Assay

Splenocytes were prepared from treated mice and resuspended in Roswell Park Memorial Institute (RPMI) 1640 medium supplemented with 10% fetal bovine serum (FBS), 50 U/mL penicillin, 50 mg/mL streptomycin, and 0.5 mg/mL amphotericin B. 2 × 10^5^ cells/well of splenocytes were treated with Con A or LPS for 72 h in an innate immune experiment, and treated with Con A, LPS, or OVA for 72 h in an adaptive immune experiment. Cell proliferation was measured by OD490 using the CellTiter 96 Aqueous One Solution Cell Proliferation Assay (Promega). Results were expressed as the stimulation index, and the formula for calculating stimulation index is shown below:

Stimulation index = OD490 of Con A, LPS, or OVA-stimulated cells/OD490 of un-stimulated cells.

### 3.8. NK Cell Activity Aassay

NK cell activity was measured by the release of the fluorescent marker from YAC-1 target cells (A lymphoma which was induced by inoculation of the Moloney leukemia virus (MLV) into a newborn A/Sn mouse) [American Type Culture Collection (ATCC) TIB-160, Food Industry Research and Development Institute, Hsinchu, Taiwan]. Splenocytes (effector cells, including NK cells) were cultured in the supplemented RPMI 1640 medium. YAC-1 cells were incubated with 2′-7′-bis (carboxyethyl)-5(6)-carboxyfluorescein (BCECF) (ThermoFisher) for 30 min at 37 °C. After being washed with culture medium, cells were re-suspended in the medium and cultured for 2 h at 37 °C. 2′,7′ –bis-(Carboxyethyl)-5-(and-6)-carboxyfluorescein (BCECF)-labeled YAC-1 cells were mixed with splenocytes as effector cells to make a 10 and 25 ratio of effector cells to target cells (E/T). After incubation for 4 h, the cultured supernatant was harvested and measured fluorescence with excitation and emission wavelengths of 485 nm and 538 nm. The total target cell fluorescence was determined by lysis of cells with 0.25% (*v*/*v*) Triton X-100. Spontaneous fluorescence was measured from target cells incubated without effector cells. Test group fluorescence was measured from the effector cell mixing with target cells. Results were expressed as NK cell activity, and the formula for calculating NK cell activity is shown below:

NK cell activity = (test group fluorescence-spontaneous fluorescence)/(total target cell fluorescence- spontaneous fluorescence) × 100%.

### 3.9. Phagocytic Activity Assay by Peritoneal Cells

Mice were scarified and peritoneal cells were harvested by injecting 5 mL of Hank’s balanced salt solution (HBSS) into the peritoneal cavity and gently shaking it. Then, peritoneal fluid was collected and centrifuged at 450× *g*, 4 °C for 10 min. The cell pellet was resuspended at 1 × 10^6^ cells/mL in supplemented RPMI 1640 medium. The phagocytosis was performed by monitoring the engulfment of FITC-labeled *E. coli* by the peritoneal exudate cells. Cells were mixed with FITC-*E. coli* at Multiplicity of infection (MOI) of 12.5 and 25 for 30 min. Fluorescence from non-internalized FITC-*E. coli* was quenched by the addition of acidified trypan blue, and the cells were then washed and analyzed by flow cytometry (BD FACSAria, Becton Dickinson Biosciences, San Jose, CA, US). Results were expressed as phagocytic activity, and the formula for calculating phagocytic activity is shown below:

Phagocytic activity = FITC positive peritoneal cells/total peritoneal cells × 100%.

### 3.10. Cytokines Secretion Assay

Cytokines including IL-2, IL-4, IL-5, TNF-α, and IFN-γ were measured by using enzyme-linked immunosorbent assay (ELISA) kits (R&D Systems Inc., Minneapolis, MN, USA) according to the manufacturer’s instructions. Splenocytes were prepared from treated mice and resuspended in supplemented RPMI 1640 medium. Cells were incubated at 1 × 10^6^ cells/well with 25 μg/mL OVA for 72 h in the second experiment for adaptive immune assay. After 72 h incubation, the cell-free supernatants were collected, centrifuged 450× *g*, 4°C for 10 min, and cytokines were measured by using the ELISA kits.

### 3.11. Serum Immunoglobulins Assay

In the second experiment for the adaptive immune assay, blood was centrifuged at 450× *g*, 4 °C for 10 min, and serum samples were collected for OVA-specific anti-IgG, OVA-specific anti-IgA, and OVA-specific anti-IgE. OVA-specific antibodies were detected by an indirect ELISA (Bethyl Laboratories). Results were expressed as ELISA units (EU), and the formula for calculating EU is shown below: EU = (OD450 of sample- OD_450_nm of blank)/(OD450 of positive- OD450 of blank). A positive OD450 indicated a serum with a high titer of OVA-specific antibodies [52]. In the third experiment, serum samples were collected for serum immunoglobulins assay by using a mouse immunoglobin quantitation set (Bethyl Laboratories) to measure the concentrations of IgM, IgG, and IgA, respectively.

### 3.12. Statistical Analysis

Numerical data are presented as means ± standard deviation. The data was analyzed by a one-way analysis of variance (ANOVA) and Student’s *t*-test.

## 4. Conclusions

In this study, we investigated the immune modulatory effects of orally administrated LMW fucoidan on the innate immune, adaptive immune, and MP antigen-stimulated immune response. Our results provided evidence that LMW fucoidan could increase splenocyte proliferation, NK cell activity, and phagocytic activity, as well as IL-2, IL-4, and IFN-γ secretion. In addition, LMW fucoidan enhanced antigen-specific antibody production in OVA- and MP antigen-stimulated mice. Our hope is that LMW fucoidan, a natural food supplement, could enhance immune responses, be needed for immunopotentiation, and attenuate the *M. pneumoniae* infectious disease.

## Figures and Tables

**Table 1:** Structure characteristics of fucoidan fractions isolated from the *L. japonica*.

	Elution Volume (mL)	Yield (%)	Sulfate (%)	Neutral Monosaccharide (% mol)						
				Fucose	Glucose	Galactose	Myo-Inositol	Mannose	Xylose	Rhamnose
Fraction 1	No. 100–115	16.1 ± 1.5	10.6 ± 0.6	12.9 ± 0.2	20.6 ± 0.2	35.8 ± 0.5	6.2 ± 0.5	17.5 ± 0.1	3.6 ± 0.1	3.4 ± 0.1
Fraction 2	No. 120–140	40.8 ± 1.2	25.2 ± 0.5	34.6 ± 0.4	25.2 ± 0.5	11.0 ± 0.7	3.6 ± 0.3	21.7 ± 0.6	1.9 ± 0.1	2.0 ± 0.2
Fraction 3	No. 150–165	34.9 ± 2.0	39.5 ± 0.8	48.2 ± 0.3	3.8 ± 0.2	26.7 ± 0.3	1.6 ± 0.4	18.4 ± 0.6	0.4 ± 0.2	0.9 ± 0.1
LMW fucoidan	NA	NA	31.4 ± 1.6	40.5 ± 0.8	5.7 ± 0.7	28.3 ± 0.8	5.4 ± 0.5	15.6 ± 0.5	3.3 ± 0.6	1.2 ± 0.4

NA, not applicable.

**Table 2:** Proliferative response of splenocytes from mice treated with LMW fucoidan.

	Stimulation Index ^‡^	
	Con A	LPS
Control	4.24 ± 0.66 ^a^	1.78 ± 0.07 ^a^
200 mg/kg	4.85 ± 0.74 ^b^	1.95 ± 0.07 ^b^
600 mg/kg	4.91 ± 0.45 ^b^	1.93 ± 0.09 ^b^
1000 mg/kg	4.92 ± 0.51 ^b^	1.96 ± 0.10 ^b^

^‡^ Stimulation index was expressed as OD490 of Con A, LPS, or OVA-stimulated cells/OD490 of unstimulated cells. Data were expressed as mean ± SD of ten mice, and analyzed using one-way ANOVA followed by Duncan’s multiple range test. Values with different letters in the same column were significantly different (*p* < 0.05).

**Table 3:** Natural killer (NK) cell activity of splenocytes from mice treated with LMW fucoidan.

	NK Cell Activity ^‡^	
	E/T Ratio ^#^: 10	E/T Ratio: 25
Control	14.4 ± 3.8 ^a^	33.1 ± 1.8 ^a^
200 mg/kg	20.8 ± 7.6 ^b^	42.1 ± 1.6 ^b^
600 mg/kg	21.0 ± 7.1 ^b^	41.4 ± 1.7 ^b^
1000 mg/kg	21.1 ± 4.4 ^b^	42.0 ± 1.9 ^b^

^‡^ NK cell activity was expressed as (test group fluorescence − spontaneous fluorescence)/(total target cell fluorescence − spontaneous fluorescence) × 100%. ^#^ Effector cell (splenocytes) to target cell (YAC-1 cell) ratio. Data were expressed as mean ± SD of ten mice, and analyzed using one-way ANOVA followed by Duncan’s multiple range test. Values with different letters in the same column were significantly different (*p* < 0.05).

**Table 4:** Phagocytic activity of peritoneal cells from mice treated with LMW fucoidan.

	Phagocytic Activity ^‡^ (%)	
	MOI 12.5	MOI 25
Control	20.1 ± 3.6 ^a^	28.7 ± 6.2 ^a^
200 mg/kg	22.9 ± 3.0 ^ab^	32.4 ± 4.4 ^ab^
600 mg/kg	23.7 ± 4.4 ^ab^	36.4 ± 5.0 ^b^
1000 mg/kg	24.8 ± 3.8 ^bc^	37.4 ± 5.6 ^b^

^‡^ Phagocytic activity was expressed as FITC-positive peritoneal cells/total peritoneal cells × 100%. Data were expressed as mean ± SD of ten mice, and analyzed using one-way ANOVA followed by Duncan’s multiple range test. Values with different letters in the same column were significantly different (*p* < 0.05).

**Table 5:** Proliferative response by ConA, LPS, or OVA stimulated splenocytes from OVA-immunized mice treated with LMW fucoidan.

	Stimulation Index ^‡^		
	Con A	LPS	OVA
Control	3.63 ± 0.56 ^a^	1.52 ± 0.25 ^a^	0.81 ± 0.03 ^a^
OVA-immunized	3.86 ± 0.27 ^a^	1.81 ± 0.09 ^a^	1.10 ± 0.03 ^b^
200 mg/kg-OVA	4.96 ± 0.28 ^b^	2.25 ± 0.25 ^c^	1.54 ± 0.04 ^c^
600 mg/kg-OVA	5.02 ± 0.23 ^b^	2.44 ± 0.18 ^c^	1.54 ± 0.03 ^c^
1000 mg/kg-OVA	5.05 ± 0.29 ^b^	2.75 ± 0.24 ^c^	1.60 ± 0.04 ^c^

^‡^ Stimulation index was expressed as OD490 of Con A, LPS, or OVA-stimulated cells/OD490 of unstimulated cells. Data were expressed as mean ± SD of ten mice, and analyzed using one-way ANOVA followed by Duncan’s multiple range test. Values with different letters in the same column were significantly different (*p* < 0.05).

**Table 6:** Cytokines secretion by OVA-stimulated splenocytes from OVA-immunized mice treated with LMW fucoidan.

	Unstimulated Basal Level	Mitogen Stimulation OVA
	**IL-2 (pg/mL)**	
Control	13.31 ± 2.40 ^a^	193.8 ± 59.9 ^a^
OVA-immunized	13.67 ± 1.44 ^a^	219.8 ± 12.1 ^a^
200 mg/kg-OVA	14.48 ± 3.66 ^a^	299.9 ± 16.6 ^b^
600 mg/kg-OVA	14.58 ± 2.70 ^a^	368.0 ± 88.7 ^b^
1000 mg/kg-OVA	14.51 ± 2.76 ^a^	538.4 ± 122.3 ^c^
	**IL-4 (pg/mL)**	
Control	3.36 ± 0.31 ^a^	5.09 ± 0.64 ^a^
OVA-immunized	3.61 ± 0.29 ^b^	9.91 ± 0.42 ^b^
200 mg/kg-OVA	3.43 ± 0.28 ^b^	9.36 ± 0.61 ^b^
600 mg/kg-OVA	3.45 ± 0.29 ^b^	13.18 ± 0.59 ^c^
1000 mg/kg-OVA	3.27 ± 0.25 ^b^	14.23 ± 0.48 ^c^
	**IL-5 (pg/mL)**	
Control	1.00 ± 0.50 ^a^	5.07 ± 0.64 ^a^
OVA-immunized	2.66 ± 0.15 ^b^	9.91 ± 0.47 ^b^
200 mg/kg-OVA	2.62 ± 0.13 ^b^	9.36 ± 0.92 ^b^
600 mg/kg-OVA	2.47 ± 0.13 ^b^	9.30 ± 0.99 ^b^
1000 mg/kg-OVA	2.56 ± 0.24 ^b^	9.41 ± 1.07 ^b^
	**IFN-γ (pg/mL)**	
Control	1.68 ± 0.23 ^a^	1014.90 ± 38.52 ^a^
OVA-immunized	51.13 ± 1.14 ^b^	1628.13 ± 32.26 ^b^
200 mg/kg-OVA	51.62 ± 2.86 ^b^	1934.48 ± 39.83 ^c^
600 mg/kg-OVA	50.91 ± 2.80 ^b^	1944.67 ± 86.95 ^c^
1000 mg/kg-OVA	51.66 ± 2.40 ^b^	1975.95 ± 44.42 ^c^
	**TNF-α (pg/mL)**	
Control	6.46 ± 0.96 ^a^	9.08 ± 1.62 ^a^
OVA-immunized	6.49 ± 0.35 ^a^	21.12 ± 3.23 ^c^
200 mg/kg-OVA	6.37 ± 0.64 ^a^	16.46 ± 2.14 ^b^
600 mg/kg-OVA	6.39 ± 0.46 ^a^	15.25 ± 0.84 ^b^
1000 mg/kg-OVA	6.31 ± 0.68 ^a^	15.02 ± 1.45 ^b^

Data were expressed as mean ± SD of ten mice, and analyzed using one-way ANOVA followed by Duncan’s multiple range test. Values with different letters in the same column were significantly different (*p* < 0.05).

**Table 7:** Serum immunoglobulins levels of OVA-immunized mice treated with LMW fucoidan.

	Serum Immunoglobulin (EU ^‡^)		
	Anti-OVA IgG	Anti-OVA IgA	Anti-OVA IgE
Control	0.04 ± 0.01 ^a^	0.06 ± 0.01 ^a^	0.02 ± 0.01 ^a^
OVA-immunized	2.12 ± 0.78 ^b^	0.96 ± 0.03 ^b^	0.85 ± 0.29 ^c^
200 mg/kg-OVA	2.39 ± 0.46 ^bc^	1.05 ± 0.07 ^b^	0.84 ± 0.11 ^c^
600 mg/kg-OVA	2.61 ± 0.24 ^c^	1.93 ± 0.04 ^c^	0.57 ± 0.07 ^b^
1000 mg/kg-OVA	2.77 ± 0.42 ^c^	2.21 ± 0.08 ^c^	0.37 ± 0.12 ^b^

^‡^ EU was expressed as (OD450 of sample − OD450 of blank)/(OD450 of positive- OD450 of blank). Data were expressed as mean ± SD of ten mice, and analyzed using one-way ANOVA followed by Duncan’s multiple range test. Values with different letters in the same column were significantly different (*p* < 0.05).
